# Atrial stretch and arrhythmia after myocardial infarction

**DOI:** 10.18632/aging.101729

**Published:** 2018-12-28

**Authors:** Olympia Bikou, Changwon Kho, Kiyotake Ishikawa

**Affiliations:** 1 Cardiovascular Research Center, Icahn School of Medicine at Mount Sinai, New York, NY 10029, USA

**Keywords:** atrial arrhythmia, arrhythmogenicity, expansion, wall stretch

Aging predisposes patients to various types of cardiovascular diseases. A recent update on Heart Disease and Stroke Statistics [1] reported an average of one heart attack in every 40 seconds for Americans. Despite the fact that therapies targeting acute coronary syndrome significantly improved over the past few decades, cardiovascular disease remains the leading cause of death: it accounts for 1 out of every 3 deaths in the US. One of the dilemmas associated with this is that patients who survived an acute coronary event develop another cardiovascular problem, eventually leading to death. Therefore, in addition to primary treatments for acute coronary syndrome, prevention of secondary cardiovascular diseases also requires attention.

One of the most frequent comorbidities after acute coronary syndrome is atrial fibrillation (AF). Incidence of AF in patients after myocardial infarction (MI) is frequent and ranges between 6 and 21% [2]. AF increases the risk of heart failure while heart failure increases AF, thus forming a positive feedback loop. AF can also increase the incidence of thromboembolic stroke with an increased risk in patients with higher age, hypertension, diabetes, and heart failure [3]. Not surprisingly, post-MI AF is associated with increased risk of death. This trend is particularly prominent in elderly patients [4].

In contrast to the primary AF where pulmonary vein isolation is effective, there are no established therapies against AF that complicates acute and subacute MI. This is partly because we currently do not fully understand the mechanisms of increased AF susceptibility at this phase. Multiple possible mechanisms have been proposed to be responsible including, inflammation, neurohormonal activation, atrial stretch, and atrial infarction. Unfortunately, no therapies against these pathologies have yet shown clear efficacy in clinical AF therapy.

In a recent preclinical study [5], we reported the importance of left atrial (LA) stretch in AF induction as well as AF maintenance in a pig model of subacute MI. Elevated left ventricular (LV) end-diastolic pressure after MI in pigs was accompanied by an increase in both LA pressure and LA volume. To examine LA physiology during altered LV loading condition, a catheter-based LV assist device, Impella, and an LA pressure-volume catheter were inserted into the LA in a closed-chest setting. These unique catheter-based experimental approaches allowed us to avoid influence of pressure changes associated with open-chest and open-pericardium procedures in this low-pressure chamber. We observed a reduction of both pressure and volume of LA by mechanical LV unloading with Impella. Importantly, reduction of LA pressure and volume reduced the incidence of pacing induced AF. Moreover, mechanical LV unloading shortened the time to spontaneous termination of induced AF, indicating that arrhythmia maintenance was also suppressed.

The analysis of LA pressure-volume relationship during the mechanical LV unloading indicated that the LA became more compliant compared to the pre-LV unloading condition. Since a change in LA tissue composition during acute LV unloading (-2 hours) is unlikely, improved LA compliance is most likely because of the alleviated LA stretch. Our interpretation is that elevated LA pressure post-MI stretches LA wall and makes it difficult to expand further. Unloading of LV can reduce LV end-diastolic pressure. This reduces LA pressure and alleviates LA stretch, thus leading to improved compliance. Stretch increases the surface area of the LA, which may prolong the conduction of electrical activity that goes through the LA and facilitates arrhythmia initiation and maintenance. Meanwhile, reversal of stretch may diminishe this stretch-mediated arrhythmic substrate. Interestingly, analysis of LA tissues from our animals indicated that stretch-dependent oxidative stress was increased in the LA after MI, whereas it was reduced in animals those underwent mechanical LV unloading. This was also associated with reduced phosphorylation of ryanodine receptor at both PKA dependent (S2808) and CAMKII dependent sites, supporting beneficial impact of LV unloading on LA arrhythmogenicity.

Our data therefore indicate that LA stretch can modify AF substrate and plays key roles in promotion of AF at least in a subacute MI setting. Atrial stretch may therefore be a promising target for development of new therapeutic approaches. Yet, several questions remain to be answered. These include, but not limited to, 1) whether LA stretch also plays similarly important roles in chronic development of AF, 2) whether same mechanism exist in non-ischemic cardiac disease, such as heart failure with preserved ejection fraction, and 3) if right atrial stretch also can similarly promote AF.

It is well known that AF begets AF by promoting electrical and structural remodeling [6]. This may be more exaggerated in post-MI setting where there is a strong stress on the LA. Although it remains to be proved in more chronic studies, inhibition of AF in acute phase of MI may be able to block the positive feedback loop that promotes subsequent electrical remodeling in the chronic phase. LV unloading, including the pharmacological approach, for a short period of time after MI to lower LV end-diastolic pressure and LA pressure may thus be a viable approach for reducing future secondary cardiovascular disease.
